# Antifungal susceptibility profiles of candidemia isolates on the east coast of Peninsular Malaysia and reliability of Etest compared to Sensititre YeastOne

**DOI:** 10.1128/spectrum.02170-25

**Published:** 2026-01-29

**Authors:** Siti Nur Syuhadah Abdullah, Mohd Nizam Tzar, Siti Norlia Othman

**Affiliations:** 1 Department of Medical Microbiology and Immunology, Faculty of Medicine, Universiti Kebangsaan Malaysia, Kuala Lumpur, Malaysia; University of Texas Medical Branch at Galveston, Galveston, Texas, USA

**Keywords:** candidemia, non-albicans *Candida*, antifungal susceptibility, resistance, Etest, Sensititre

## Abstract

Candidemia is a major cause of morbidity and mortality worldwide. The rising prevalence of non-albicans *Candida* species (NACs), which often show diverse antifungal susceptibility, complicates treatment. This study aimed to characterize the species distribution and antifungal susceptibility of *Candida* isolates from candidemia cases on the east coast of Peninsular Malaysia and to evaluate the reliability of Etest compared to Sensititre YeastOne (SYO). A cross-sectional study collected 143 *Candida* blood culture isolates from several hospitals on the east coast of Peninsular Malaysia between February 2019 and January 2020. Species identification was performed using conventional methods and MALDI-TOF MS. Antifungal susceptibility testing was conducted with SYO and Etest. MIC results were interpreted using CLSI and EUCAST guidelines. Agreement between methods was assessed by essential and categorical agreement and error rates. NACs predominated (82%), with *C. parapsilosis* (27.3%), *C. tropicalis* (24.5%), and *C. glabrata* (21%). Echinocandins and amphotericin B showed high susceptibility rates (96.2% and 91.5%, respectively). Azole resistance was notable, especially in *C. tropicalis* and *C. glabrata*. Etest demonstrated an inconsistent agreement with SYO, particularly for fluconazole and anidulafungin, with high major and very major error rates. NACs dominate candidemia in Malaysia’s east coast, with significant azole resistance, while echinocandins and amphotericin B remain effective. Comparative analysis demonstrated poor agreement and elevated error rates for Etest relative to SYO, particularly for azoles and anidulafungin, indicating its unreliability as a stand-alone method. Continued local surveillance and validated AFST methods are critical to guide effective antifungal therapy.

**IMPORTANCE:**

This study highlights the evolving epidemiology of candidemia in Malaysia's east coast, marked by rising dominance of drug-resistant non-albicans *Candida* species. Findings underscore the value of reliable susceptibility testing, demonstrating that the widely used Etest method may misclassify resistance status, potentially impacting treatment choices. These insights inform stewardship efforts and clinical management in resource-constrained settings.

## INTRODUCTION

Fungal infections have become an increasing concern over the past decade, with *Candida* species emerging as a significant cause of morbidity and mortality worldwide. The incidence of candidemia has remained stable or slightly increased, with approximately 7.4 cases per 100,000 population and an in-hospital mortality rate around 32.6% (1). *Candida albicans* continues to be a leading cause of bloodstream infections. However, there has been a significant increase in non-albicans *Candida* species (NACs) such as *Candida tropicalis, Candida parapsilosis,* and *Candida glabrata* both globally and in Malaysia (2–4). Echinocandins, such as anidulafungin, caspofungin, micafungin, and rezafungin, are preferred first-line treatments for candidemia due to their broad effectiveness against various *Candida* species (including *C. auris*), good safety profile, and minimal drug interactions (5). If echinocandins are not available or if resistance is present, alternatives such as liposomal amphotericin B, fluconazole, and voriconazole are moderately recommended, though their use is limited by toxicity and interactions. Other amphotericin B formulations and certain azoles (amphotericin B colloidal dispersion, itraconazole, and posaconazole) are generally discouraged when better options exist (5). Local studies in Malaysia have reported low susceptibility of *Candida* species to itraconazole, with rates around 40% (6, 7). Rising fluconazole resistance should also be taken into account. Antifungal susceptibility testing (AFST) is essential for detecting resistance and guiding effective therapy. The Clinical and Laboratory Standards Institute (CLSI) broth microdilution method remains the gold standard but is labor-intensive and requires specialized expertise, limiting its routine clinical use (8). The European Committee on Antimicrobial Susceptibility Testing (EUCAST) also provides standardized broth microdilution protocols for yeasts, with the latest version 7.4 released in 2023 (9). Alternative methods such as the Etest and Sensititre YeastOne (SYO) assays have gained widespread acceptance due to their ease of use and good correlation with reference methods. The Etest offers a user-friendly and promising gradient diffusion format but may show variability in minimum inhibitory concentration (MIC) results for azoles due to trailing growth phenomena (10). SYO utilizes colorimetric indicators facilitating accurate and reproducible MIC readings, with categorical agreement rates often exceeding 90% compared to CLSI broth microdilution across multiple *Candida* species and antifungal agents (11). Due to the labor-intensive nature of broth microdilution and the established strong agreement between SYO and CLSI reference methods, SYO was selected as a practical comparative standard to evaluate the performance of Etest in this study. Our objectives were to assess the agreement between Etest and SYO for antifungal susceptibility testing in candidemia and to characterize the species distribution and antifungal susceptibility patterns of *Candida* isolates from the east coast of Peninsular Malaysia.

## RESULTS

### Isolate collection

A total of 143 *Candida* blood culture isolates were collected at the mycology laboratory of Hospital Tengku Ampuan Afzan (HTAA) during the study period. Of these, 55 isolates (38.5%) originated from HTAA itself, while the remaining 88 isolates (61.5%) were referred from other regional hospitals. Species identification was performed, with 83 (58%) of the isolates identified by MALDI-TOF MS and the remaining 60 (42%) characterized using conventional methods. All 143 isolates underwent antifungal susceptibility testing using the SYO method. Additionally, 129 isolates were tested by the Etest method for susceptibility to amphotericin B, fluconazole, and anidulafungin.

### Species distribution

**Fig 1:**
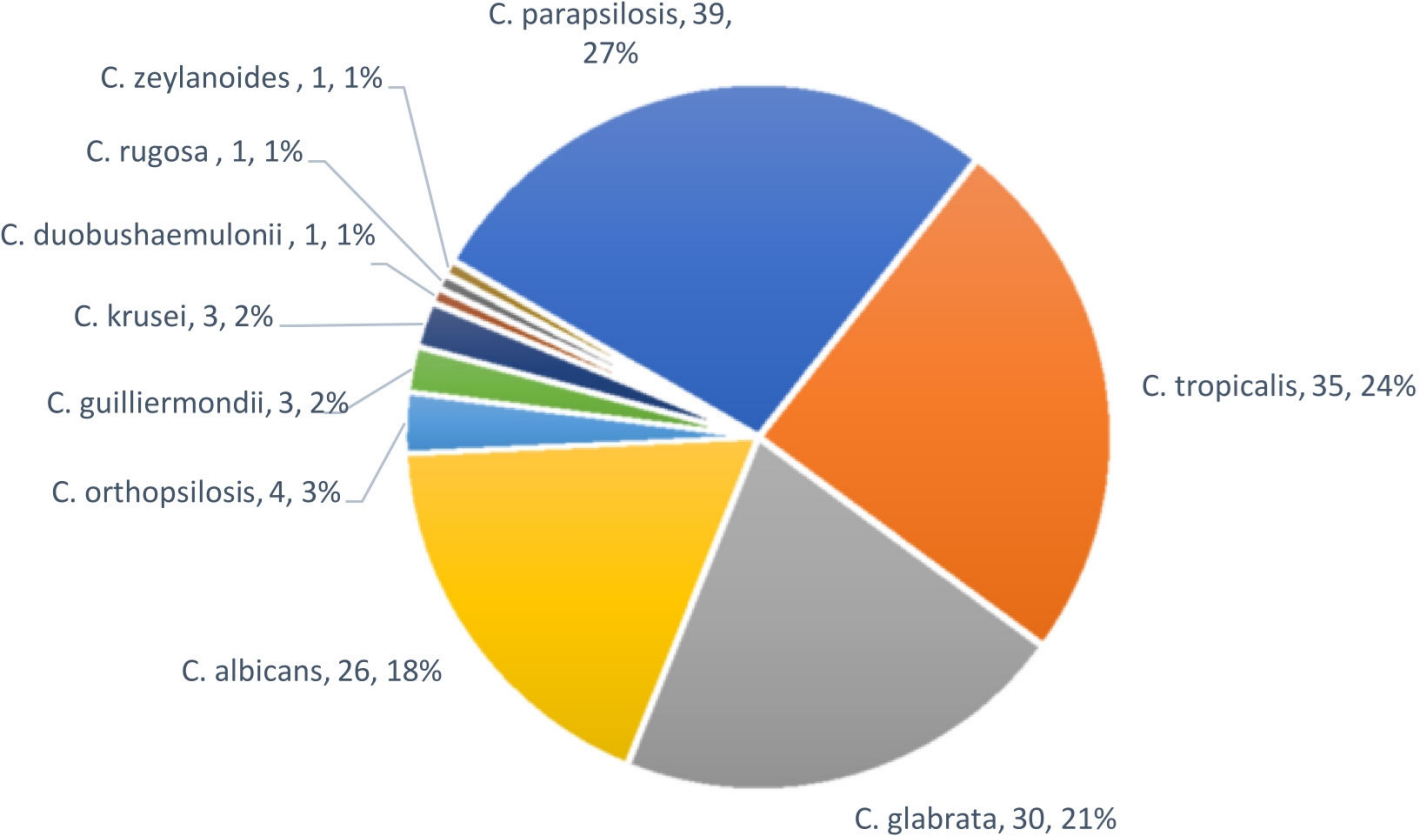
*Candida* species distribution of candidemia cases.

Among the 143 *Candida* isolates, NACs predominated, comprising 82% (117/143) of isolates, whereas *C. albicans* accounted for only 18% (26/143). The most frequently isolated NACs were *C. parapsilosis* (27.3%), *C. tropicalis* (24.5%), and *C. glabrata* (21%) (Fig. 1).

### Antifungal susceptibility profiles

**Fig 2:**
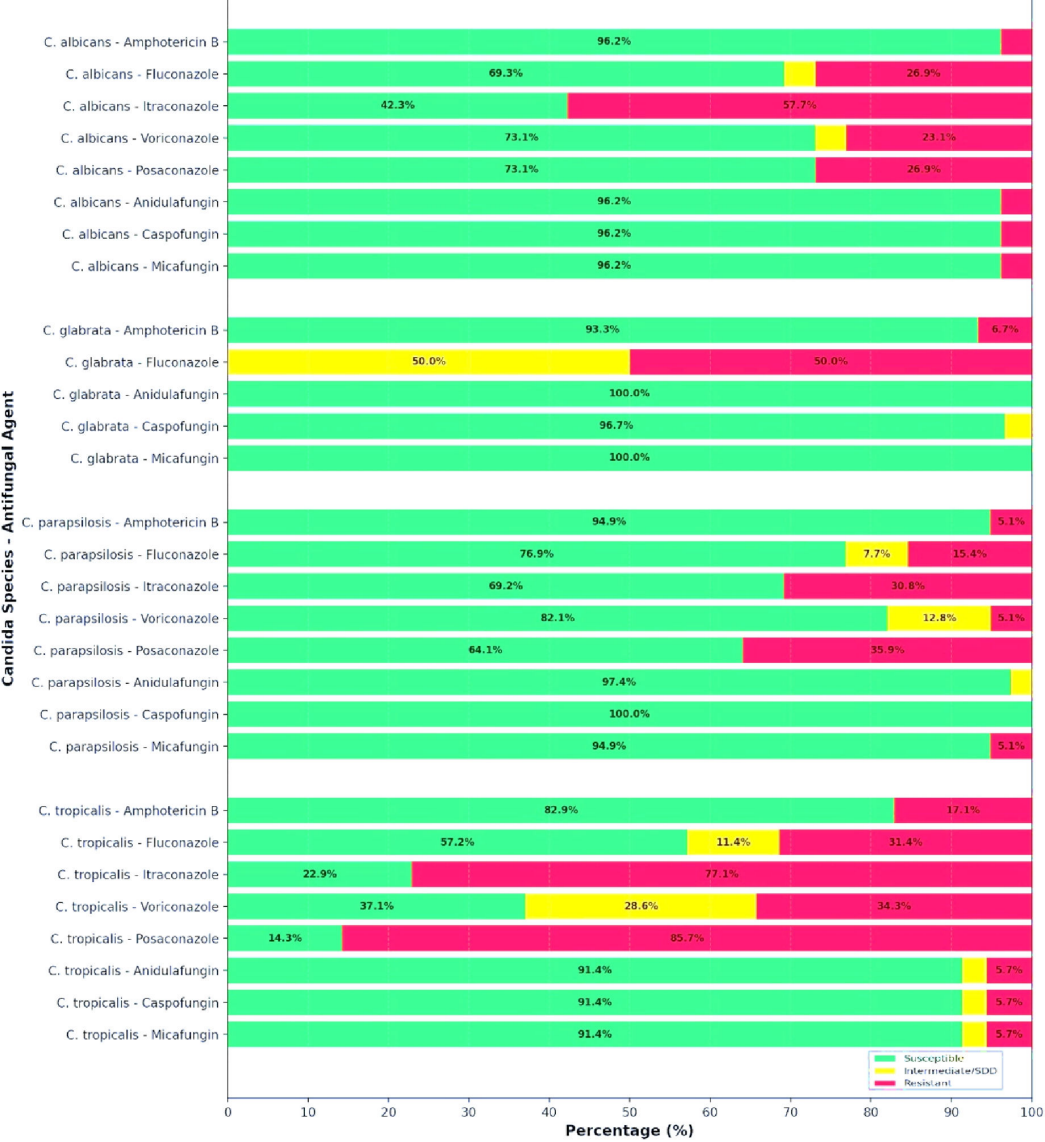
Antifungal susceptibility profiles of the four most common *Candida* species, performed using SYO and interpreted according to clinical breakpoints from CLSI M27M44S (2022) guidelines (for fluconazole, voriconazole, anidulafungin, caspofungin, and micafungin) and EUCAST version 11.0 (2024) guidelines (for amphotericin B, itraconazole, and posaconazole). Except for fluconazole, breakpoints for other azoles against *C. glabrata* are not available.

Table 1 summarizes the MIC distributions of various antifungal agents across different *Candida* species, as determined by SYO, while Fig. 2 presents their interpretations based on the CLSI M27M44S (2022) and EUCAST version 11.0 (2024) breakpoints (8, 12).

**TABLE 1:** Minimum inhibitory concentrations (MICs) of antifungal agents to blood culture isolates of *Candida* species as determined by the SYO method^*b*^

	Antifungal agent minimum inhibitory concentrations, MICs^*a*^ (µg/mL)								
	AMB	FCZ	ICZ	VCZ	PCZ	ANF	CPF	MCF	5FC
*C. parapsilosis (n = 39*)									
Range	< 0.12–2	0.12–64	0.015–16	< 0.008–2	< 0.008–2	< 0.015–4	< 0.008–1	< 0.008–4	< 0.06–4
MIC_50_	0.5	1	0.12	0.015	0.06	1	0.5	1	< 0.06
MIC_90_	1	8	0.25	0.25	0.25	2	1	2	0.25
MIC_GEO_	0.5	1.5	0.12	0.04	0.07	0.6	0.3	0.6	0.11
*C. tropicalis (n = 35*)									
Range	0.5–2	0.5 to > 256	0.06 to > 16	0.03 to > 8	0.06 to > 8	0.015 to > 8	0.015 to > 8	0.015 to > 8	< 0.06 to > 64
MIC_50_	1	2	0.25	0.25	0.25	0.12	0.06	0.03	< 0.06
MIC_90_	2	> 256	> 16	> 8	> 8	0.25	0.25	0.03	0.25
MIC_GEO_	0.9	5.6	0.5	0.43	0.43	0.14	0.08	0.03	0.09
*C. glabrata (n = 30*)									
Range	< 0.12–2	4 to > 256	0.06 to > 16	0.03 to > 8	0.12 to > 8	< 0.015–0.06	0.008–0.25	< 0.008–0.015	< 0.06–2
MIC_50_	0.5	32	1	1	2	0.03	0.03	0.015	< 0.06
MIC_90_	1	> 256	> 16	> 8	> 8	0.06	0.06	0.015	0.25
MIC_GEO_	0.6	57	2	1	2	0.03	0.04	0.012	0.09
*C. albicans (n = 26*)									
Range	0.25–2	0.25 to > 256	0.03 to > 256	< 0.008 to > 8	0.015 to > 8	0.015 to > 8	0.015 to > 8	< 0.008 to > 8	< 0.06 to > 64
MIC_50_	0.5	1	0.12	0.03	0.06	0.06	0.06	0.015	0.12
MIC_90_	1	> 256	> 256	> 8	> 8	0.12	0.12	0.015	0.25
MIC_GEO_	0.6	3.5	0.4	0.08	0.13	0.05	0.05	0.015	0.13
*C. orthopsilosis (n = 4*)									
Range	0.5–2	0.25–0.5	0.03–0.06	< 0.008–0.015	0.015–0.06	1	0.06–1	0.5–1	< 0.06–0.12
*C. guilliermondii (n = 3*)									
Range	< 0.12–0.25	2–4	0.25–0.5	0.06–0.12	0.12–0.25	0.25–1	0.06–1	0.12–0.5	< 0.06
*C. krusei (n = 3*)									
Range	0.5–2	4–64	0.5	0.25–0.5	0.25–0.5	0.015–0.12	0.06–0.5	0.03–0.12	< 0.06–16
*C. duobushaemulonii (n = 1*)									
Range	> 8	> 256	> 16	> 8	> 8	0.12	0.06	0.12	< 0.06
*C. rugosa (n = 1*)									
Range	0.25	0.25	0.03	< 0.008	0.06	1	0.25	0.5	< 0.06
*C. zeylanoides (n = 1*)									
Range	0.5	1	0.12	0.015	0.06	0.25	0.25	0.5	0.12

^*a*^ MIC_50_, MIC_90_, and geometric mean MICs were only calculated for *Candida* spp. with at least 10 isolates.

^*b*^ AMB, amphotericin B; FCZ, fluconazole; ICZ, itraconazole; VCZ, voriconazole; PCZ, posaconazole; ANF, anidulafungin; CPF, caspofungin; MCF, micafungin; 5FC, flucytosine.

*Candida parapsilosis* generally showed low MIC values for most antifungals, indicating good susceptibility. Amphotericin B had MIC_50_ and MIC_90_ values of 0.5 and 1 µg/mL, respectively. Fluconazole demonstrated some variability with an MIC_90_ of 8 µg/mL, suggesting reduced susceptibility in part of the population. Echinocandins such as anidulafungin, caspofungin, and micafungin had MIC_90_ values between 1 and 2 µg/mL, consistent with known moderately elevated MICs in this species. Other azoles, including itraconazole, voriconazole, and posaconazole, showed low MIC values, reflecting retained antifungal activity. Susceptibility rates were high for caspofungin at 100%, anidulafungin at 97.4%, micafungin at 94.9%, and amphotericin B at 94.9%, but noticeably lower for fluconazole at 76.9%, voriconazole at 82.1%, itraconazole at 69.2%, and posaconazole at 64.1%.

*Candida tropicalis* demonstrated marked resistance to several antifungals, especially azoles. The fluconazole MIC_90_ exceeded 256 µg/mL, indicating high-level resistance. MIC_90_ values for itraconazole and voriconazole also surpassed clinical thresholds of 16 and 8 µg/mL, respectively. Meanwhile, echinocandins maintained generally low MICs, with MIC_50_ values well below resistance cutoffs, suggesting that their effectiveness remains largely preserved. Amphotericin B showed moderate activity, with an MIC_50_ of 1 µg/mL, and flucytosine MICs varied broadly but remained low at MIC_50_. Susceptibility rates were 91.4% for echinocandins, 82.9% for amphotericin B, and notably lower for azoles: 57.2% for fluconazole, 37.1% for voriconazole, 22.9% for itraconazole, and 14.3% for posaconazole.

*Candida glabrata* exhibited significant azole resistance, with fluconazole MIC_90_ values above 256 µg/mL and itraconazole MIC_90_ over 16 µg/mL. The geometric mean MIC for fluconazole was high at 57 µg/mL, reflecting frequent resistance within this species. Echinocandins showed very low MICs; for example, anidulafungin had an MIC_50_ of 0.03 µg/mL, consistent with their role as preferred antifungal agents. Amphotericin B maintained moderate efficacy with an MIC_50_ of 0.5 µg/mL, although some isolates showed MICs up to 2 µg/mL. Susceptibility percentages were 100% for anidulafungin and micafungin, 96.7% for caspofungin, and 93.3% for amphotericin B. Fluconazole susceptibility was 0%, with half of the isolates classified as susceptible-dose-dependent. Breakpoints for voriconazole, itraconazole, and posaconazole are not established for *C. glabrata* (8, 12).

*Candida albicans* generally remained susceptible to amphotericin B and echinocandins, with low MIC_50_ and MIC_90_ values. However, resistance was observed for fluconazole and itraconazole, with some isolates showing MIC_90_ values exceeding 256 µg/mL. Voriconazole and posaconazole exhibited broad MIC ranges, but MIC_50_ values indicated overall susceptibility. Flucytosine MICs were generally low. Susceptibility rates were high for caspofungin, anidulafungin, micafungin, and amphotericin B at 96.2% each, moderate for voriconazole and posaconazole at 73.1%, lower for fluconazole at 69.3%, and lowest for itraconazole at 42.3%.

Other less common species—including *C. orthopsilosis, C. guilliermondii, C. krusei, C. duobushaemulonii, C. rugosa,* and *C. zeylanoides*—had limited isolate numbers and showed variable MIC ranges. Some isolates exhibited higher MICs, suggesting possible resistance concerns, especially to azoles and amphotericin B; however, conclusions are limited by small sample sizes.

**Fig 3:**
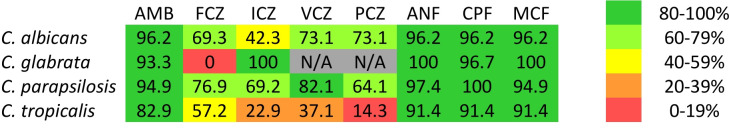
A heatmap of antifungal susceptibility (%) of the four most common *Candida* species, performed using Sensititre YeastOne and interpreted according to clinical breakpoints from CLSI M27M44S (2022) guidelines (for fluconazole, FCZ; voriconazole, VCZ; anidulafungin, ANF; caspofungin, CPF; micafungin, MCF) and EUCAST version 11.0 (2024) guidelines (for amphotericin B, AMB; itraconazole, ICZ; posaconazole, PCZ).

Overall, echinocandins and amphotericin B retained strong antifungal activity across the *Candida* species tested, supporting their continued use, particularly for empirical therapy. Azole susceptibility varied greatly, with *C. tropicalis* and *C. glabrata* showing significant resistance especially to fluconazole and itraconazole (Fig. 3). The broad MIC ranges and geometric mean MICs observed highlight intra-species differences and emphasize the importance of performing susceptibility testing to guide targeted antifungal therapy.

### Comparison of Etest and SYO

Table 2 compares the MIC_50_, MIC_90_, and geometric mean MIC values of amphotericin B, fluconazole, and anidulafungin, as measured by SYO and Etest across various *Candida* species.

**TABLE 2:** Antifungal MICs of amphotericin B, fluconazole, and anidulafungin to *Candida* spp., as determined by SYO and Etest methods^*c*^

*Candida* sp. (n) Antifungal agent	Test method	MIC range (µg/mL)	MIC_50_ (µg/mL)	MIC_90_ (µg/mL)	Geometric mean MIC
*C. parapsilosis* (38)					
Amphotericin B	SYO	0.12–2	0.5	1	0.5
	Etest	0.003–2	0.5	1	0.38
Fluconazole	SYO	0.12–64	1	8^*a*^	1.5
	Etest	0.064 to > 256	1	32^*a*^	2.05
Anidulafungin	SYO	0.015–4	1	2^*a*^	0.6
	Etest	0.003–32	1	32^*a*^	0.55
*C. tropicalis* (30)					
Amphotericin B	SYO	0.25–1	1	2	**0.9** ^*b*^
	Etest	0.002–1	0.5	1	**0.24** ^*b*^
Fluconazole	SYO	0.25 to > 256	2	**>256** ^*b*^	**5.6** ^*b*^
	Etest	0.023 to > 256	1.5	**64** ^*b*^	**1.94** ^*b*^
Anidulafungin	SYO	0.03–32	**0.12** ^*b*^	**0.5** ^*b*^	0.14
	Etest	0.004–32	**0.032** ^*b*^	**0.12** ^*b*^	0.1
*C. glabrata* (28)					
Amphotericin B	SYO	0.03–2	0.5	1	0.6
	Etest	0.016–2	0.75	2	0.55
Fluconazole	SYO	4 to > 256	**32** ^*b*^	>256	**57** ^*b*^
	Etest	0.064 to > 256	**8** ^*b*^	>256	**11.98** ^*b*^
Anidulafungin	SYO	0.015–0.06	0.03^*a*^	0.06^*a*^	0.03^*a*^
	Etest	0.006–32	0.5^*a*^	4^*a*^	0.31^*a*^
*C. albicans* (22)					
Amphotericin B	SYO	0.25–1	0.5	1	0.6
	Etest	0.004–4	0.5	2	0.4
Fluconazole	SYO	0.25 to > 256	1	**>256** ^*b*^	3.5
	Etest	0.064 to > 256	1	**128** ^*b*^	2.01
Anidulafungin	SYO	0.015–8	**0.25** ^*b*^	0.12^*a*^	0.05
	Etest	0.002–4	**0.023** ^*b*^	1.5^*a*^	0.05
*C. guilliermondii* (3)					
Amphotericin B	SYO	0.12–0.25	0.25	0.25^*a*^	0.2^*a*^
	Etest	0.38–2	0.38	2^*a*^	0.66^*a*^
Fluconazole	SYO	2–4	4	4^*a*^	3.2^*a*^
	Etest	3.0–16	6	16^*a*^	6.6^*a*^
Anidulafungin	SYO	0.25–1	1^*a*^	1^*a*^	0.6^*a*^
	Etest	1.5 to > 32	>32^*a*^	>32^*a*^	11.54^*a*^
*C. krusei* (3)					
Amphotericin B	SYO	0.5–2	1	2	1^*b*^
	Etest	0.023–2	0.75	2	**0.325** ^*b*^
Fluconazole	SYO	4 to > 256	64	>256	25.4
	Etest	4 to > 256	32	>256	32
Anidulafungin	SYO	0.015–0.12	0.06^*a*^	0.12^*a*^	0.05^*a*^
	Etest	0.032–32	4^*a*^	32^*a*^	1.6^*a*^
*C. orthopsilosis* (2)					
Amphotericin B	SYO	1–2	1.5	2	**1.4** ^*b*^
	Etest	0.38–1	0.75	3	**0.62** ^*b*^
Fluconazole	SYO	0.25–0.5	**0.5** ^*b*^	0.5	0.35
	Etest	0.19–0.5	**0.19** ^*b*^	0.5	0.3
Anidulafungin	SYO	1	1	1^*a*^	1
	Etest	0.75–3	0.75	3^*a*^	1.5
*C. duobushaemulonii* (1)					
Amphotericin B	SYO	>8	**>8** ^*b*^	**>8** ^*b*^	**>8** ^*b*^
	Etest	0.5	**0.5** ^*b*^	**0.5** ^*b*^	**0.5** ^*b*^
Fluconazole	SYO	>256	**>256** ^*b*^	**>256** ^*b*^	**>256** ^*b*^
	Etest	4	**4** ^*b*^	**4** ^*b*^	**4** ^*b*^
Anidulafungin	SYO	0.12	0.12^*a*^	0.12^*a*^	0.12^*a*^
	Etest	12	12^*a*^	12^*a*^	12^*a*^
*C. rugosa* (1)					
Amphotericin B	SYO	0.25	0.25^*a*^	0.25^*a*^	0.25^*a*^
	Etest	1	1^*a*^	1^*a*^	1^*a*^
Fluconazole	SYO	0.25	0.25^*a*^	0.25^*a*^	0.25^*a*^
	Etest	2	2^*a*^	2^*a*^	2^*a*^
Anidulafungin	SYO	1	1^*a*^	1^*a*^	1^*a*^
	Etest	8	8^*a*^	8^*a*^	8^*a*^
*C. zeylanoides* (1)					
Amphotericin B	SYO	0.25	0.25^*a*^	0.25^*a*^	0.25^*a*^
	Etest	1	1^*a*^	1^*a*^	1^*a*^
Fluconazole	SYO	1	1^*a*^	1^*a*^	1^*a*^
	Etest	4	4^*a*^	4^*a*^	4^*a*^
Anidulafungin	SYO	0.25	0.25^*a*^	0.25^*a*^	0.25^*a*^
	Etest	1	1^*a*^	1^*a*^	1^*a*^

^*a*^ Etest MIC is higher than SYO more than one double-dilution (Etest overestimation).

^*b*^ Etest MIC is lower than SYO more than one double-dilution (Etest underestimation).

^*c*^ Bold values indicate underestimation of MICs by Etest compared to SYO. Such underestimation may cause MICs to be incorrectly interpreted as less resistant or even susceptible, which could adversely affect treatment outcomes.

For *C. parapsilosis*, amphotericin B MICs were consistent between SYO and Etest, indicating reliable results. Fluconazole MICs showed some variation, with Etest generally reporting higher MIC_90_ values, which suggests it may overestimate resistance. Anidulafungin MICs agreed well at the MIC_50_ level but showed more variability at higher MICs, with Etest tending to overestimate.

In *C. tropicalis*, amphotericin B susceptibility remained strong, although Etest reported slightly lower MICs. Fluconazole MIC_90_ values differed significantly between methods, with SYO indicating very high resistance levels that Etest somewhat underestimated. Anidulafungin MICs were variable across the two methods, showing only partial agreement and demonstrating inconsistency in Etest readings.

For *C. glabrata*, amphotericin B MICs were similar between the two methods. Fluconazole MICs revealed substantial differences, with SYO detecting higher resistance. Anidulafungin MICs were low by SYO but often higher and more variable by Etest, suggesting possible overestimation and inconsistent results.

*Candida albicans* showed good agreement between SYO and Etest for amphotericin B MIC_50_ values, though Etest reported a slightly higher MIC_90_. For fluconazole, MIC_50_ values were the same between methods, but Etest recorded a lower MIC_90_ than SYO, suggesting slight underestimation of high-level resistance by Etest. Anidulafungin MICs differed significantly, with Etest underestimating the MIC_50_ compared to SYO by more than one dilution.

Among the rare *Candida* species such as *C. guilliermondii, C. krusei, C. orthopsilosis, C. duobushaemulonii, C. rugosa, and C. zeylanoides*, MICs varied widely between SYO and Etest. Discrepancies were especially notable for fluconazole and anidulafungin, with Etest sometimes overestimating or underestimating resistance compared to SYO. The small sample sizes in this group limit definitive conclusions.

Overall, amphotericin B MICs showed good agreement between SYO and Etest across *Candida* species, supporting reliable susceptibility testing for this drug. In contrast, fluconazole and anidulafungin MICs demonstrated variable agreement depending on the species. Notable discrepancies occurred in *C. tropicalis, C. albicans,* and *C. glabrata*, with Etest frequently overestimating or underestimating MICs, especially at higher values. These findings indicate that although Etest offers a convenient alternative, results for azoles and echinocandins should be interpreted with caution and confirmed by reference methods whenever possible to ensure accurate and effective antifungal therapy.

Table 3 compares Etest antifungal susceptibility results to those from SYO across four *Candida* species, evaluating EA, CA, mE, ME, and VME.

**TABLE 3:** Essential agreement (EA), categorical agreement (CA), and error rates of Etest as compared to SYO^*b*^,

Candida species (n)	Parameter (%)^*a*^	Amphotericin B	Fluconazole	Anidulafungin
*Candida parapsilosis (38*)	EA	73.7	63	39.5
	CA	89.5	84.2	68.4
	mE	NA^*c*^	10.5	18.4
	ME	7.9	**2.6**	13.2
	VME	2.6	2.6	**0**
*Candida tropicalis (30*)	EA	53.3	43.3	22.7
	CA	76.7	66.7	60
	mE	NA	**10**	**6.7**
	ME	3.3	10	26.7
	VME	20	13	6.7
*Candida glabrata (28*)	EA	53.6	32.1	21.4
	CA	57.1	60.7	42.9
	mE	NA	39.3	**0**
	ME	39.3	**0**	57.1
	VME	3.6	**0**	**0**
*Candida albicans (22*)	EA	77.3	36.4	22.7
	CA	**90.9**	72.7	77.3
	mE	NA	**9.1**	13.6
	ME	4.5	9.1	9.1
	VME	4.5	9.1	**0**

^*a*^ mE, minor error; ME, major error; VME, very major error.

^*b*^ Boldface values indicate acceptable EA and CA (≥90%) and acceptable mE (≤10%), ME (≤3%), and VME (≤1.5%)%.

^*c*^ N/A indicates not applicable.

For amphotericin B, although CA was fairly high in *C. albicans* (90.9%) and *C. parapsilosis* (89.5%), EA was below 90% for all species. Moreover, MEs exceeded the acceptable 3% limit in several species, such as 7.9% in *C. parapsilosis* and 4.5% in *C. albicans*, while VMEs surpassed the 1.5% threshold in both species, indicating unacceptable error rates and raising concerns about reliability.

Fluconazole showed poor performance with EA and CA well below 90% across all species. mEs were unacceptably high, especially in *C. glabrata* (39.3%), and VMEs were notably elevated in *C. tropicalis* (13%) and *C. albicans* (9.1%), posing a significant risk of falsely categorizing resistant isolates as susceptible.

Anidulafungin showed the weakest performance with low EA (as low as 21.4%) and CA (42.9% in *C. glabrata*). MEs greatly exceeded acceptable levels in *C. glabrata* (57.1%) and *C. tropicalis* (26.7%), emphasizing frequent misclassification by Etest despite low VMEs.

In summary, none of the antifungal agents tested with Etest met all acceptable criteria, with amphotericin B showing concerning error rates despite moderate CA. Fluconazole and anidulafungin results were particularly unreliable, marked by low agreement and high error rates. These findings highlight that Etest should be used cautiously and confirmed with reference methods to ensure accurate antifungal susceptibility assessment.

## DISCUSSION

This study provides important insights into the epidemiology and antifungal susceptibility of *Candida* species isolated from blood cultures in Pahang and Terengganu, Peninsular Malaysia, over 1 year. NACs accounted for 82% of candidemia cases, predominantly *C. parapsilosis, C. tropicalis,* and *C. glabrata,* whereas *C. albicans* represented 18%. This distribution aligns with findings from a study on candidemia in Kelantan, another east coast state, where *C. parapsilosis* is the predominant species (13). In contrast, *C. albicans* is more common in Kuala Lumpur (7). These differences emphasize regional variability in species prevalence and highlight the critical need for localized surveillance to inform empirical antifungal therapy. Globally, *C. glabrata* is most commonly found in North America, while *C. parapsilosis* is more prevalent in Latin America, Europe, and Africa. Countries in Asia and Oceania report relatively higher incidences of both *C. glabrata* and *C. tropicalis* (2).

High azole resistance rates were observed among common species, with itraconazole (41.5%), posaconazole (39.2%), and fluconazole (30%) resistance notably elevated. Resistance in *C. albicans* to fluconazole reached 26.9%, markedly higher than observed in a previous Malaysian report (<5%), aligning with global trends (14). Molecular mechanisms underpinning this resistance include ERG11 mutations that reduce drug binding affinity, overexpression of ERG11, and upregulation of efflux pumps such as CDR1, CDR2, and MDR1, all contributing to elevated MICs and clinical treatment failure (15). Additionally, fluconazole resistance is linked to enhanced virulence phenotypes through modulation of biofilm formation, hyphal growth, adhesins, and proteases (16). Voriconazole resistance emerged notably in *C. tropicalis* (34.3%) and *C. albicans* (23.1%), likely driven by overlapping resistance mechanisms, including biofilm-mediated drug tolerance (17).

Echinocandins remain the recommended first-line treatment for candidemia (5), but resistance rates in this study were higher than observed in earlier Malaysian study data: micafungin resistance of 5.1% in *C. parapsilosis*, 5.7% in *C. tropicalis*, and 3.9% in *C. albicans*, with no resistance detected in *C. glabrata* (4, 7). These resistances are most probably associated with point mutations in the FKS1 gene, which reduce the binding affinity of the antifungal to its target enzyme (17). Although genotypic confirmation of these mutations was not performed here due to resource constraints, the observed phenotypes align with global data emphasizing the need for ongoing molecular surveillance to inform clinical management.

Amphotericin B has shown an emerging trend of resistance compared to previous reports in Malaysia (4, 6, 14). Potential mechanisms underlying this resistance include biofilm formation and mutations affecting the ergosterol synthesis pathway (17). Despite these concerns, amphotericin B demonstrated the highest concordance between SYO and Etest methods, reinforcing its continued role as a valuable alternative treatment for resistant infections, albeit with consideration of its known toxicity risks.

Our comparative analysis of antifungal susceptibility testing methods identified significant limitations of Etest, especially for fluconazole and anidulafungin, with poor EA and CA noted predominantly in *C. glabrata* and *C. tropicalis*, respectively. The high rates of VMEs (false susceptibility) with Etest are clinically concerning as these may lead to inappropriate therapy and treatment failure, particularly in resource-limited settings where Etest is often the sole available method. This underscores the risk of relying solely on Etest for guiding antifungal therapy.

Etest trailing growth with azoles in *Candida* species, particularly *C. albicans, C. glabrata*, and *C. tropicalis*, is characterized by persistent microcolonies within the inhibition ellipse that complicate MIC reading. The paradoxical effect seen with echinocandins involves enhanced growth at high drug concentrations within the inhibition zone but does not indicate true resistance (10). Both phenomena present challenges for precise antifungal susceptibility interpretation using Etest compared with the more reproducible, colorimetric broth microdilution-based SYO (11). The latter also benefits from earlier reliable readings (as soon as 24 hours), minimal operator bias, and closer alignment with CLSI reference standards, making it the preferred method in clinical laboratories.

While previous literature recognizes Etest as a promising method requiring further evaluation (10), our data clearly demonstrate its current limitations in accurately detecting resistance in key antifungal classes, especially azoles and echinocandins. This finding cautions against uncritical adoption of Etest in clinical decision-making without confirmatory testing.

In summary, this study highlights the shifting epidemiology of candidemia in Malaysia, with NAC predominance and rising azole and echinocandin resistance posing treatment challenges. It reinforces the critical need for regional antifungal susceptibility monitoring, integration of molecular diagnostics where feasible, and careful interpretation of Etest results to avoid compromising patient outcomes. A graphical summary of resistance profiles would enhance data accessibility and support clinical translation. These findings carry significant implications for antifungal stewardship and policy, particularly in resource-constrained settings relying on Etest for susceptibility guidance.

This study has several limitations. Notably, there is a temporal gap between the study period (February 2019 to January 2020) and the year of publication (2025). This delay resulted from the extensive time required for detailed data analysis and manuscript preparation, compounded by resource and staffing constraints. Additionally, disruptions caused by the COVID-19 pandemic affected clinical and laboratory workflows, contributing further to the elapsed time. The timing of manuscript submission was strategically aligned with the release of updated antifungal susceptibility guidelines and related research developments to enhance the study’s relevance. Although this gap may limit the immediate applicability of the findings in rapidly evolving epidemiological contexts, the data still provide valuable insights into regional candidemia trends and antifungal resistance patterns. Another important limitation is the unavailability of patient clinical demographic data, which restricts the ability to correlate microbiological findings with clinical outcomes and risk factors. Furthermore, genotypic confirmation of antifungal resistance mechanisms was not performed in the current study. Given the complexity and polygenic nature of antifungal resistance involving mutations in target genes such as ERG11 and FKS1, as well as efflux pump regulation, future investigations incorporating molecular and genomic approaches are warranted to better elucidate resistance mechanisms and support phenotypic findings. Lastly, the relatively small sample size compared to larger multicenter studies may limit the generalizability of findings; therefore, further multicenter investigations with larger cohorts are recommended to confirm and expand these results.

### Conclusions

The predominance of NACs with diverse antifungal susceptibility profiles in Malaysia underscores the necessity for routine species-level identification and susceptibility testing to optimize candidemia management. Echinocandins and amphotericin B remain the most effective antifungal agents against the majority of bloodstream *Candida* isolates. High azole resistance, particularly in *C. tropicalis* and *C. glabrata*, limits their empirical use and highlights the critical importance of antifungal stewardship programs. The Etest method demonstrated unreliable performance compared to SYO, particularly for fluconazole and anidulafungin. Therefore, it should be used with caution and not as a standalone test, and results are best confirmed by validated reference or commercial susceptibility testing methods. Continuous surveillance and updated local susceptibility data are essential to guide treatment protocols and monitor emerging resistance trends.

## MATERIALS AND METHODS

### Study design and population

A 1-year cross-sectional study was conducted from February 2019 to January 2020 at Hospital Tengku Ampuan Afzan (HTAA), a regional referral center that processes candidemia samples from multiple hospitals on the east coast of Peninsular Malaysia. All *Candida* isolates from blood cultures were included, excluding duplicate isolates and those considered to be line colonizers.

### Diagnostic criteria

Candidemia was confirmed by isolating *Candida* species from one or more peripheral blood cultures in patients presenting with clinical signs of bloodstream infection, in accordance with standard diagnostic criteria (5).

### Identification of yeast isolates

From February to June 2019, *Candida* species identification was performed using a combination of germ tube tests, CHROMagar, slide culture, and API 20C systems. Starting in July 2019, identification primarily relied on MALDI-TOF MS, which improved both accuracy and turnaround time.

### Antifungal susceptibility testing

Susceptibility testing was conducted using SYO colorimetric microdilution for amphotericin B, anidulafungin, caspofungin, micafungin, fluconazole, itraconazole, voriconazole, posaconazole, and flucytosine and Etest gradient diffusion method for amphotericin B, fluconazole, and anidulafungin, following the manufacturers’ instructions (see Supplementary Materials at https://doi.org/10.5281/zenodo.17946818) (18, 19). Minimum inhibitory concentration (MIC) interpretations were based on breakpoints established by CLSI M27M44S (8) guidelines (for fluconazole, voriconazole, and echinocandins) and EUCAST version 11.0 (12) (for amphotericin B, itraconazole, and posaconazole). Since breakpoints for flucytosine are not yet established by either organization, its MIC data were not interpreted.

### Comparison of Etest and SYO

Antifungal MICs determined by Etest and SYO were recorded as MIC range, MIC_50_, MIC_90_, and geometric mean MIC. Subsequently, essential agreement (EA), categorical agreement (CA), and rates of minor errors (mEs), major errors (MEs), and very major errors (VMEs) were calculated and compared between the two methods. Detailed definitions of these terms are provided in the Supplementary Materials at https://doi.org/10.5281/zenodo.17946818 (20, 21).

### Statistical analysis

Data on *Candida* species distribution and antifungal susceptibility patterns were analyzed descriptively. Frequencies and distributions were expressed as percentages. EA, CA, and error rates between Etest and SYO were calculated and reported as percentages.
